# Activation of Platelet-Derived Growth Factor Receptor Alpha Contributes to Liver Fibrosis

**DOI:** 10.1371/journal.pone.0092925

**Published:** 2014-03-25

**Authors:** Brian J. Hayes, Kimberly J. Riehle, Masami Shimizu-Albergine, Renay L. Bauer, Kelly L. Hudkins, Fredrik Johansson, Matthew M. Yeh, William M. Mahoney, Raymond S. Yeung, Jean S. Campbell

**Affiliations:** 1 Department of Pathology, University of Washington, Seattle, Washington, United States of America; 2 Department of Pharmacology, University of Washington, Seattle, Washington, United States of America; 3 Department of Surgery, University of Washington, Seattle, Washington, United States of America; 4 Center for Cardiovascular Biology and Institute for Stem Cell and Regenerative Medicine, University of Washington, Seattle, Washington, United States of America; Medical University Graz, Austria

## Abstract

Chronic liver injury leads to fibrosis, cirrhosis, and loss of liver function. Liver cirrhosis is the 12th leading cause of death in the United States, and it is the primary risk factor for developing liver cancer. Fibrosis and cirrhosis result from activation of hepatic stellate cells (HSCs), which are the primary collagen producing cell type in the liver. Here, we show that platelet-derived growth factor receptor α (PDGFRα) is expressed by human HSCs, and PDGFRα expression is elevated in human liver disease. Using a green fluorescent protein (GFP) reporter mouse strain, we evaluated the role of PDGFRα in liver disease in mice and found that mouse HSCs express PDGFRα and expression is upregulated during carbon tetrachloride (CCl_4_) induced liver injury and fibrosis injection. This fibrotic response is reduced in *Pdgfrα* heterozygous mice, consistent with the hypothesis that liver fibrosis requires upregulation and activation of PDGFRα. These results indicate that *Pdgfrα* expression is important in the fibrotic response to liver injury in humans and mice, and suggest that blocking PDGFRα–specific signaling pathways in HSCs may provide therapeutic benefit for patients with chronic liver disease.

## Introduction

Chronic liver injury is a major cause of morbidity and mortality in the US and worldwide, due to complications of liver fibrosis, cirrhosis, and hepatocellular carcinoma (HCC) [1]. To date, there are no effective treatments for patients with liver fibrosis, so a better understanding of pathways that regulate fibrosis has great clinical potential [2]. Many inflammatory cytokines and growth factors are released during liver injury, including platelet derived growth factors (PDGFs), which are potent mitogens for hepatic stellate cells (HSCs) [2], [3]. The PDGF family of ligands and receptors plays a central role in repair after injury, and are key regulators of the formation of connective tissue [4], [5]. Elevated platelet-derived growth factor receptor (PDGFR) expression is detected in human heart disease, pulmonary fibrosis, and kidney fibrosis [6]–[8], and blocking PDGFR signaling decreases collagen deposition after myocardial infarct, in pulmonary fibrosis, and in kidney fibrosis [9]–[11]. Thus, targeting the PDGF pathway may modulate liver fibrosis.

There are five known functional ligand dimers in the PDGF family, -AA, -AB, -BB, -CC, and –DD, which bind cell surface receptor tyrosine kinases comprised of PDGFRα and PDGFRβ subunits [12]. PDGFs stimulate the migration and proliferation of mesenchymal cells during development [13]. Loss of PDGFRs leads to significant abnormalities in mice [14], [15]. PDGFRβ is critical to vascular and hematopoietic development, and cell specific deletion or activation of PDGFRβ results in failure or increased pericyte and vascular smooth muscle cell coverage of blood vessels in mice [14], [16], [17]. PDGFRα is required for migration and survival of neural crest cells and for skeletal development, and cell specific deletion of PDGFRα decreases β-cell proliferation in the pancreas and ventricular septation of the heart [15], [18], [19]. Constitutive activation of PDGFRα causes fibrosis that is particularly noticeable in intestine, skin, muscle and heart, but activation has to be conditionally induced in late prenatal or adult animals, as constitutive PDGFRα activation causes lethality [5]. Deleting one allele of *Pdgfrα* in mice does not affect development, unlike the observed phenotype in homozygous knockout mice [15], [20]. PDGF signal transduction pathways play a prominent role in fibrosis [21]. It has been suggested that PDGFRα signaling is more likely to induce fibrosis than PDGFRβ [22], however this notion has not been conclusively demonstrated in the liver. In summary, PDGF signaling is tightly regulated by abundance and degree of signal transduction, and perturbing either results in developmental defects and organ dysfunction.

In the present study we analyzed *PDGFR* in human liver disease, human liver cell lines, and a mouse model of liver injury and fibrosis. We found increased PDGFRα in human liver specimens with fibrosis and cirrhosis. PDGFRα is primarily expressed in HSCs, and *Pdgfrα* expression increased in injured mouse livers. We investigated the role of PDGFRα in liver fibrosis using mice with only one allele of *Pdgfrα*, and found that reducing *Pdgfrα* copy number inhibits liver fibrosis in mice. Together our data suggest that PDGFRα inhibitors could be an effective means to reduce liver fibrosis in patients.

## Materials and Methods

### Animals

Mice were housed in a specific pathogen-free environment overseen by the Department of Comparative Medicine at the University of Washington with IACUC approval under protocol #4295-01. Mice that express nuclear localized green fluorescent protein (GFP) driven by the endogenous *Pdgfrα* promoter, *Pdgfrα^nGFP^*, were purchased from the Jackson Laboratory (007669) [20]. Either wild type (WT) littermates that retain both *Pdgfrα* alleles, or control mice, *i.e.* male C57BL/6 mice purchased from the Jackson Laboratory (000664), were used as experimental controls. To induce fibrosis, mice were injected (*i.p*.) with 10 μl/g body weight CCl_4_ (Sigma-Aldrich) diluted in olive oil 10% (v/v), either one time (acute injury) or twice weekly for four or six weeks (chronic injury). Olive oil-injected animals served as controls for CCl_4_-injected mice. Animals were sacrificed using CO_2_ inhalation. The Institutional Animal Care and Use Committee of the University of Washington, which is certified by the Association for Assessment and Accreditation of Laboratory Animal Care International, approved all experiments.

### Human Liver Samples

Human liver and HCC specimens were obtained from the University of Washington Medical Center after IRB-approval. IHC was performed on liver specimens from patients with cirrhosis who underwent liver transplantation surgery at the University of Washington Medical Center from 1989 to 2002, HSD #23602 (MMY) [23]. Immunoblot analysis was performed on resected liver specimens collected after receiving informed consent IRB #31281 (RSY). All samples were de-identified of any patient information. Specimens were either fixed in formalin or frozen at −80°C until use.

### Immunohistochemistry (IHC) and Histological Staining

Formalin-fixed liver tissue was processed and embedded in paraffin using standard protocols, and IHC was performed as previously described [24], using the primary antibodies listed in Table S1. A board-certified clinical liver pathologist (MMY) reviewed all human samples and determined the presence of cirrhosis and/or tumor and assessed for PDGFRα and PDGFRβ immunoreactivity. To quantify fibrosis, formalin-fixed liver tissue was stained with picrosirius red. For morphometric analysis, picrosirius red area was imaged under polarized light [25]. Images were analyzed using NIH image J software to convert pixels to binary values and determine the relative number of positive and negative pixels.

### Immunoblotting

Tissues were homogenized in a 1% Triton-x 100 lysis buffer and processed as described [26]. Membranes were incubated with primary antibodies overnight at 4°C, and then with the appropriate horseradish peroxidase (HRP)-conjugated secondary antibodies. Primary antibodies used in this study are listed in Table S1.

### Immunofluorescence (IF) and *ex-vivo* Imaging

Livers were fixed in 4% paraformaldehyde overnight, and tissues were frozen in optimum cutting temperature compound for cryosectioning. IF was performed using standard techniques, with liver sections incubated overnight with the primary antibodies listed in Table S1. Immune complexes were detected with goat Alexa 633 conjugated anti-rat IgG (A-21094, Life Technologies) and goat Alexa 546 conjugated anti-rabbit IgG (A-11010, Life Technologies) antibodies. Sections were mounted with SlowFade Gold (S36936, Life Technologies) and imaged with a Leica SL confocal microscope (Leica Microsystems, Keck Center UW). For *ex-vivo* imaging, freshly harvested livers were analyzed as previously described [27]. Images were captured using a Zeiss 510 Meta confocal microscope. In *Pdgfrα^WT/nGFP^* mice, GFP fluorescence was used to report PDGFRα positive cells [20].

### Cell Culture and Proliferation Assay

Liver cell lines (Table S2) were grown in a 37°C incubator with 95% humidity and 5% CO2 in DMEM (Life Technologies) with 10% FBS. Confluent cells were split and allowed to attach to plates as described [28]. Cells were serum-starved for 24 hours then stimulated with 10 ng/ml PDGF-AA, -AB,-BB, or –CC (R&D systems) for 24 hours. [^3^H]Thymidine (1 μCi/ml final concentration) was added to the media for the final 3 hours of stimulation. Unincorporated [^3^H] thymidine was removed from the cells, and trichloroacetic acid was used to precipitate protein-bound DNA. DNA was solubilized in NaOH, quantified using a scintillation counter, and measured in triplicate.

### RNA Expression Analysis

RNA was extracted from cells or liver tissue using Trizol (15596-018, Life Technologies) as described by the manufacturer. Reactions contained cDNA synthesized from 0.5 μg RNA using MMLV (28025-013, Life Technologies), and Taqman Universal Mastermix II (4440040, Life Technologies). Cycling conditions were 95°C for 10 min, and 49 cycles of 95°C for 15 sec, 60°C for 60 sec with a final extension at 72°C for 1 min. Data are represented as delta delta Ct values after normalization to *Gapdh* mRNA levels. Primers used in this experiment are listed in Table S3.

### Statistical Analysis

Statistical significance was analyzed using Prism software (Graphpad), either with Kruskal-Wallis non-parametric ANOVA with significance p<0.05, or Mann-Whitney U test with significance p<0.05, as indicated in the figure legends.

## Results

### Expression of PDGFRα in Human Cirrhosis and HCC

**Figure 1:**
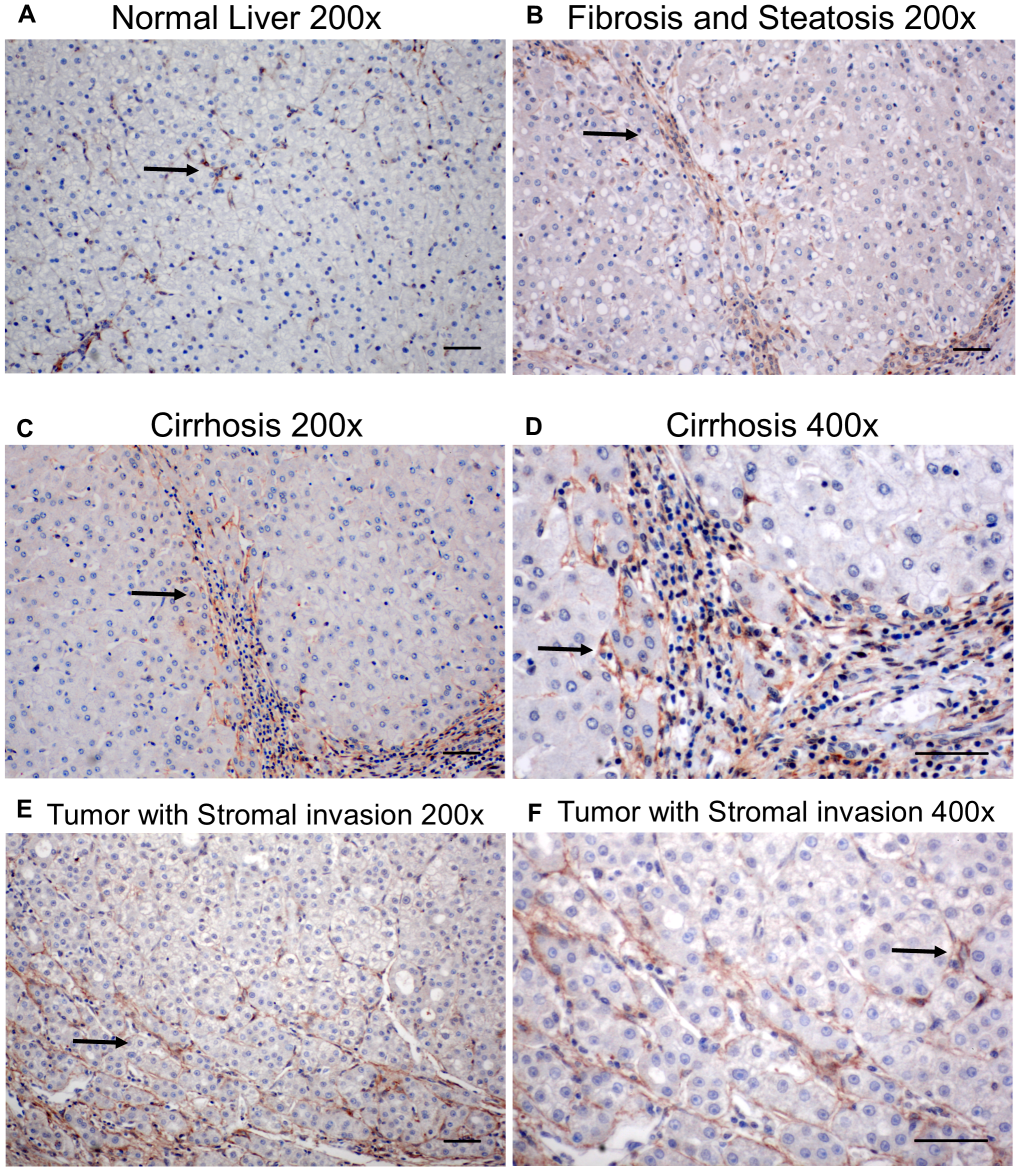
Perisinusoidal PDGFRα expression is localized to fibrotic or cirrhotic areas in tumor specimens by IHC. A) Uninjured (non-diseased) human liver demonstrate focal PDGFRα immunoreactivity (arrow) in NPCs but not hepatocytes. Resected tumor specimens show PDGFRα positive cells within fibrotic areas (arrow) (B), and cirrhotic areas (C), with sinusoidal PDGFRα immunoreactivity (arrows) within fibrotic septa (D). E and F) A resected tumor specimen shows stromal PDGFRα immunoreactivity (arrows). All scale bars are 50 μm.

Previous studies have demonstrated that over expression of PDGF ligands induces fibrosis in mice [29]–[31], and elevated expression of *PDGFRβ* in chronic liver disease has been reported [32]–[34]. As PDGF ligands can activate both PDGFRα and PDGFRβ, we sought to investigate the role of PDGFRα in chronic liver injury. 80–90% of human HCC arise in the setting of a cirrhotic liver, in which HSCs have been activated [35], so we first performed IHC analysis to determine whether PDGFRα and PDGFRβ levels are elevated in human cirrhosis and HCC. Fibrotic and cirrhotic livers had focal perisinusoidal immunoreactivity for PDGFRα, which was stronger in steatotic and cirrhotic livers, while normal adult liver had relatively little PDGFRα immunoreactivity (Figure 1 A–D). PDGFRβ immunoreactivity was also increased in the fibrotic and cirrhotic areas compared to un-injured liver (data not shown). Table S4 summarizes PDGFRα and PDGFRβ immunoreactivity in diseased human liver specimens, 77% of which demonstrated increased PDGFRα immunoreactivity and 56% of which demonstrated increased PDGFRβ immunoreactivity. Using a separate set of specimens, we compared PDGFR protein levels in grossly dissected HCC tumors to those of adjacent non-tumor livers from the same patients by immunoblot analysis. PDGFRα protein was frequently detected in the non-tumor tissue (Figure S1, Table S5). One specimen, patient 5, had detectable PDGFRα protein by immunoblot in the tumor (Table S5). IHC analysis of this specimen demonstrated PDGFRα immunoreactive cells within the tumor (Figure 1 E, F), but these cells did not have the histological appearance of hepatocytes, suggesting that non-parenchymal cells (NPCs) had invaded the parenchymal tumor and account for the PDGFRα immunoreactivity observed in this tumor by immunoblot analysis. Taken together, our data suggest that PDGFRα is expressed primarily in fibrotic and cirrhotic livers, predominantly in NPCs.

### Expression of PDGFRα and PDGFRβ in Human Liver and Stellate Cell Lines

**Figure 2:**
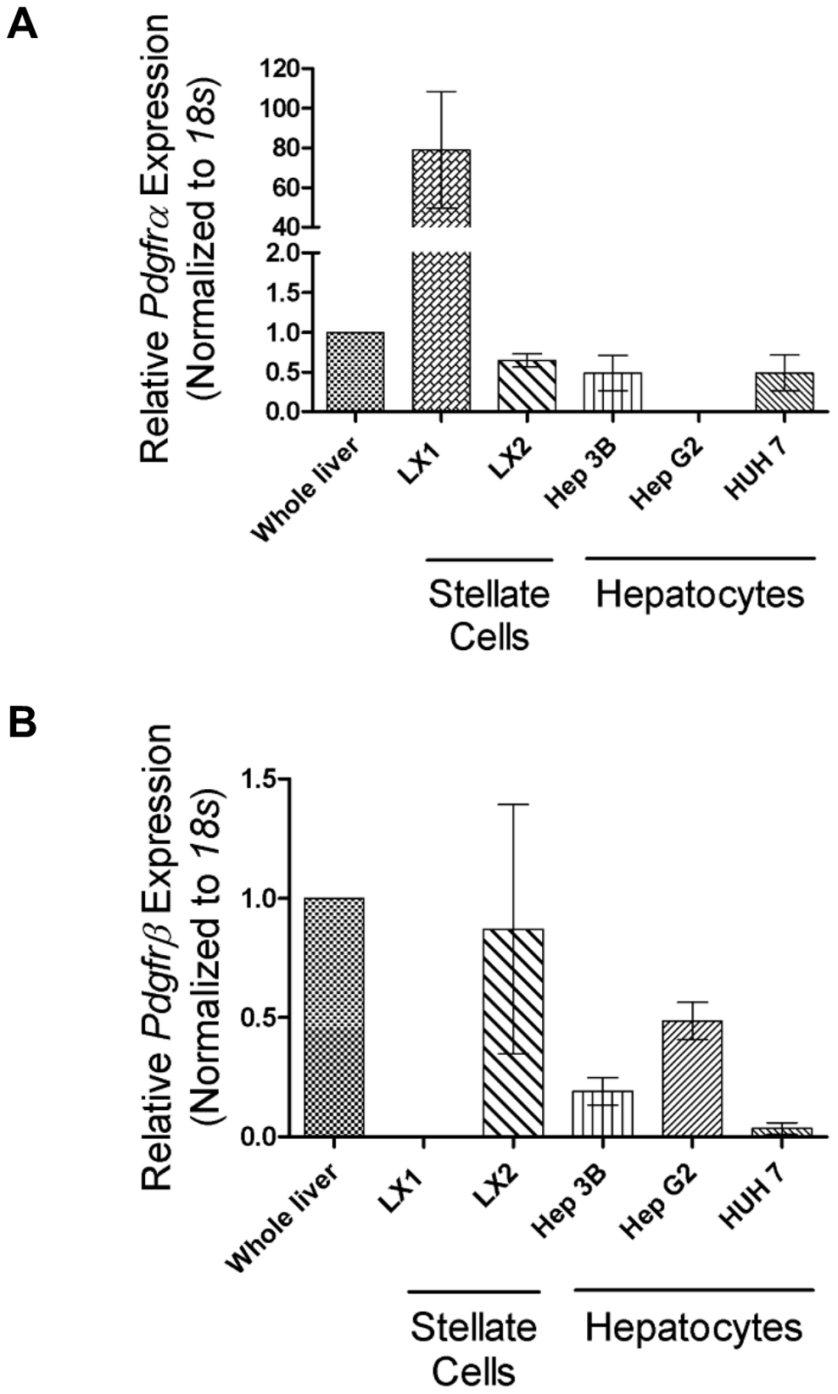
PDGF receptors are expressed in hepatic stellate cell lines. A) Relative *PDGFRα* mRNA expression is greater in LX1 HSCs compared to LX2 and hepatocyte cell lines. B) *PDGFRβ* mRNA expression in LX2 HSC is variable, but similar to whole liver and hepatocyte cell lines. *PDGFR* expression was normalized to 18S ribosomal RNA and reported as fold increase by the ΔΔCt method, normalized to adult human liver. Error bars indicate standard error of the mean, n = 3 separate cultures.

We next analyzed mRNA transcripts from human liver cell lines, and found that both *PDGFRα* and *PDGFRβ* mRNA are expressed in non-diseased human liver (Figure 2), but that the human HSC line LX-1 has a significantly higher relative expression of *PDGFRα* (Figure 2A). LX-2 cells, a LX-1 subclone, express *PDGFRβ,* albeit to variable levels, and LX-1 cells express little to no *PDGFRβ* (Figure 2B). Transcription of both PDGFRs is reduced in human hepatoma cell lines compared to whole liver, suggesting that PDGFRs are predominantly expressed in NPCs. We next stimulated various cell lines with PDGF ligands, and found that PDGF -AA, -AB, -BB, and –CC lead to robust proliferation in stellate cells, but these ligands had little effect on the hepatocyte or hepatoma cell lines tested (Table S6).

### Increased Expression of *Pdgfrα* and *Pdgfrβ* in Mice after CCl_4_ induced Hepatocyte Injury

**Figure 3:**
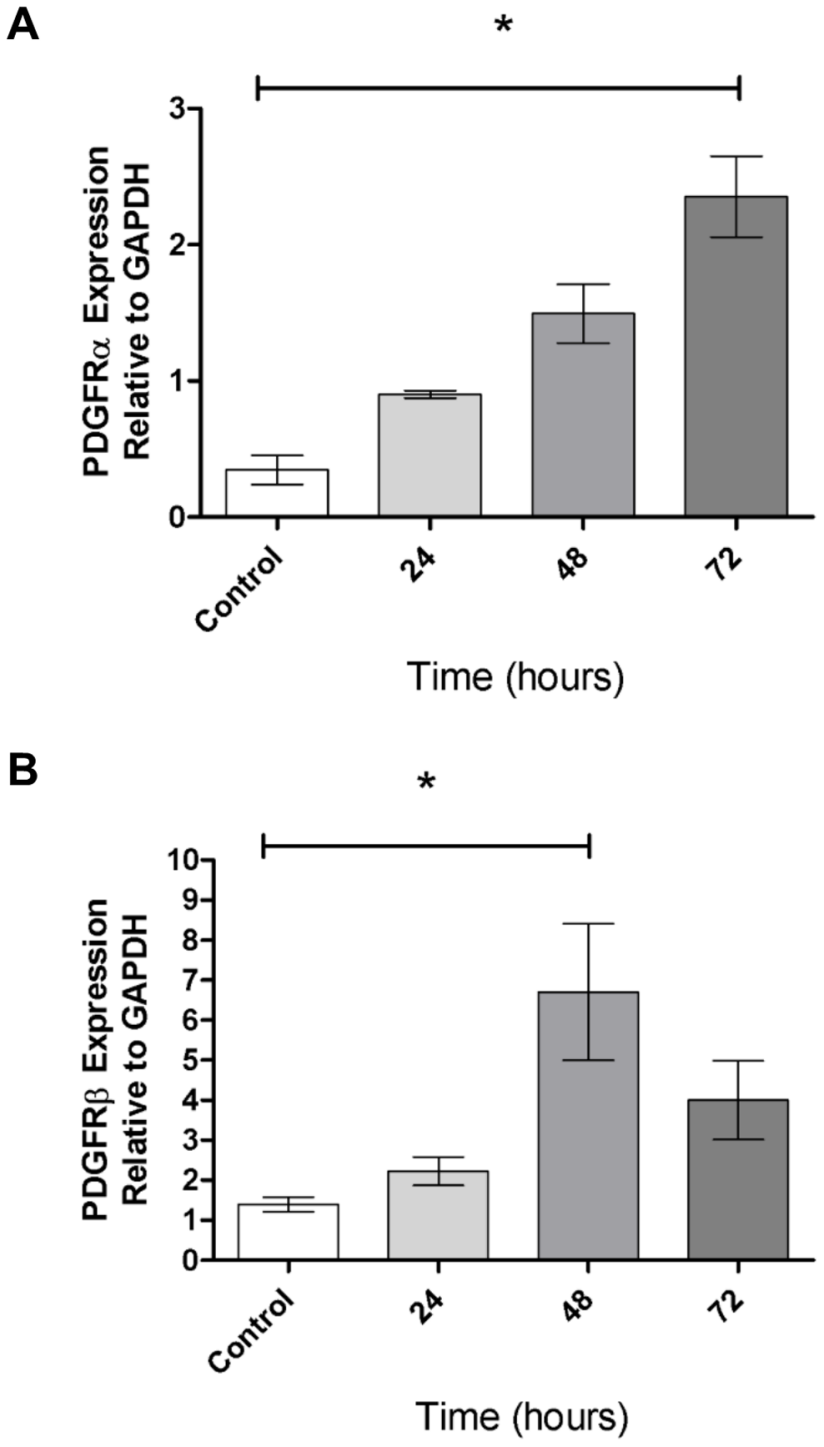
PDGFR mRNA expression increases in response to acute CCl_4_ exposure. Expression of *Pdgfrα* (A) and *Pdgfrβ* (B) increases after a single injection of CCl_4_ in C57BL/6 mice. Values are represented as means with SEM; n = 3 mice per group; and data was analyzed by Kruskal-Wallis non-parametric ANOVA * = p<0.05.

To investigate the role of PDGFRα in liver injury and fibrosis, we used the well-established model of CCl_4_ injection, in which HSCs are activated in response to necroinflammatory injury to hepatocytes [36]. CCl_4_ injury to rats has been shown to induce *Pdgfrs* mRNA in the liver [37]. To determine whether *Pdgfr* expression is induced after liver injury in mice, WT mice were injected with a single dose of CCl_4_. We found that *Pdgfrα* expression increased during 72 hours after injury (Figure 3A). CCl_4_ injection also induced expression of *Pdgfrβ*, although to a differing extent and with a different time course than *Pdgfrα* (Figure 3B). Thus, acute CCl_4_ exposure induces *Pdgfr* expression in the liver.

### Cells Expressing Pdgfrα and Pdgfrβ Respond to CCl_4_ Injury

**Figure 4:**
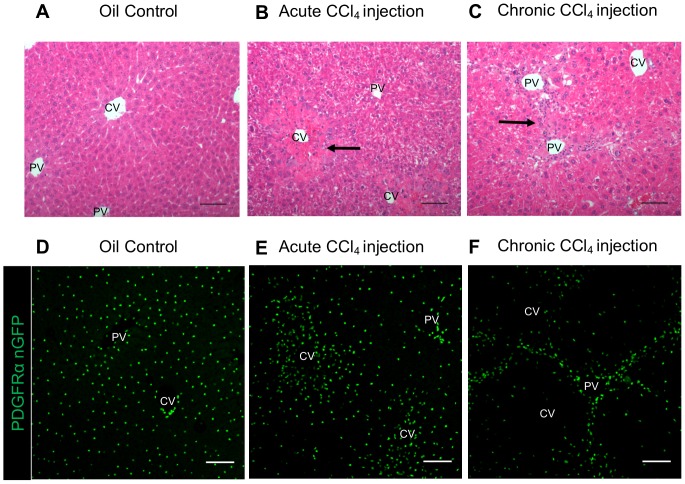
PDGFRα positive cells form fibrotic bands after chronic CCl_4_ injection in *Pdgfrα^WT/nGFP^* mice. Oil injection A) does not lead to necrosis around central veins (CV), while areas of necrosis are visible (arrow) 72 hrs after CCl_4_ injection (B) as determined by H&E. C) NPCs are visible in areas between portal veins (PV, arrow). D) PDGFRα positive cells (green nuclei) are evenly distributed throughout the liver after oil injection. E) PDGFRα positive cells localize around the CV 72 hr after CCl_4_ injury. F) PDGFRα positive cells align with fibrotic bands that develop between portal triads after chronic CCl_4_ injury. Scale bars are 100 μm.

**Figure 5:**
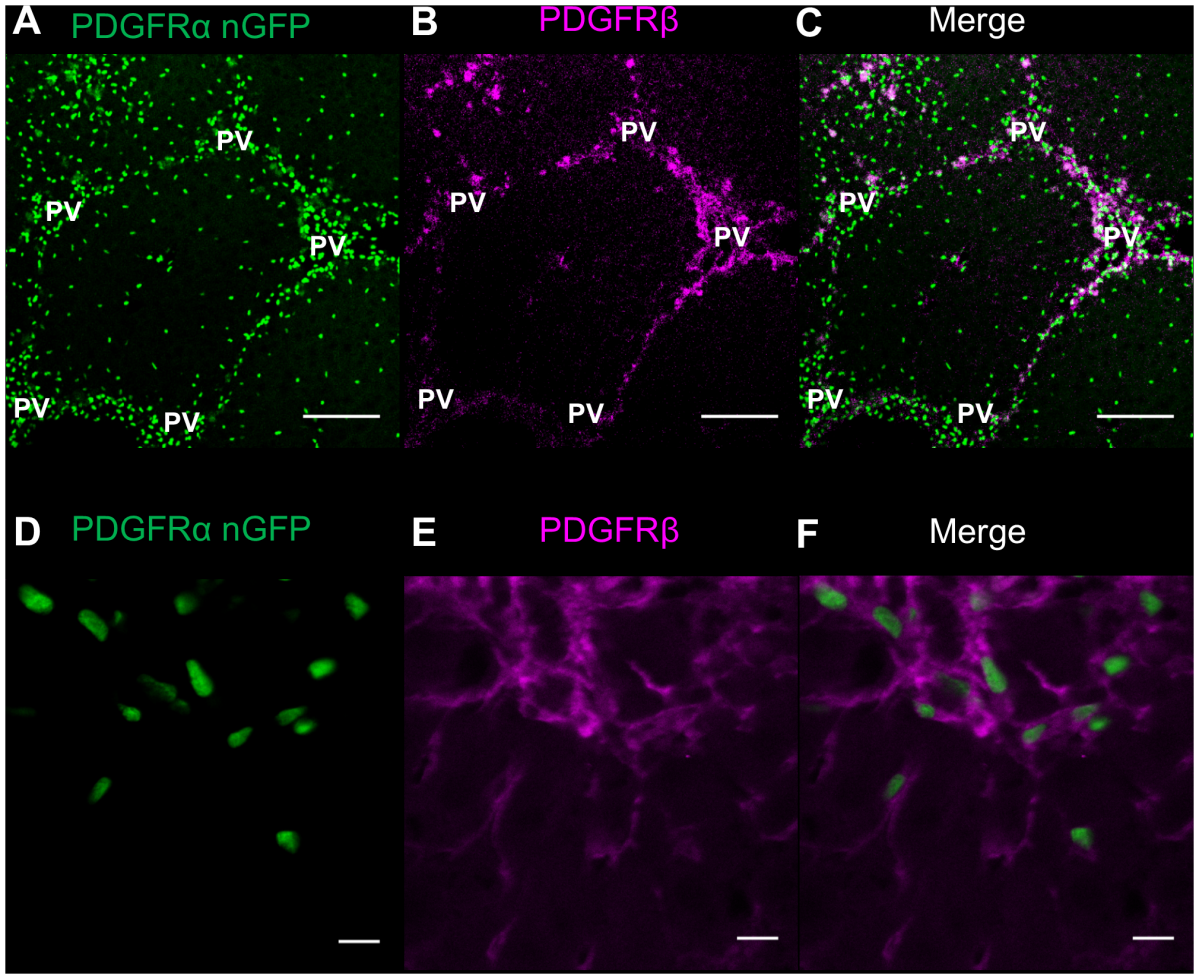
PDGFRα-positive cells co-localize with PDGFRβ-positive cells in chronic CCl_4_ injured liver. *Pdgfrα^WT/nGFP^* mice were injected with CCl_4_ twice weekly for six weeks. PDGFRα-expressing cells are identified by nuclear-localized GFP (green). PDGFRβ-expressing cells are identified by IF (PDGFRβ; magenta). A) PDGFRα-positive cells are aligned between portal veins (PV). B) PDGFRβ is expressed in the same periportal area as PDGFRα-positive cells, as shown in the merged image (C). A–C) Scale bars are 100 μm. D–F) Higher magnification shows that PDGFRα and PDGFRβ co-localize in the same cell, based upon co-localization of the GFP and PDGFRβ signal. Scale bars are 10 μm.

To determine the liver cell type that expresses PDGFRα in response to liver injury, we used transgenic *Pdgfrα^WT/nGFP^* mice, in which the endogenous *Pdgfrα* promoter initiates transcription of nuclear-restricted GFP reporter in place of the *Pdgfrα* gene [20]. These mice have one copy of *Pdgfrα* replaced by GFP and are thus heterozygous for *Pdgfrα*. While a single injection of CCl_4_ induces necrosis and injury that is repaired within seven days, repeated injection of CCl_4_ induces liver fibrosis [36]. Vehicle-injected *Pdgfrα^WT/nGFP^* mice have histologically normal liver (Figure 4A). A single CCl_4_ injection increases the density of small cells with a high nuclear to cytoplasmic ratio, suggestive of inflammatory cells, around central veins at 72 hours, while chronic injection increases the density of cells between portal veins (Figure 4B, C). As shown in Figure 4D, vehicle-injected *Pdgfrα^WT/nGFP^* mice have an even distribution of PDGFRα-positive cells throughout the liver lobule. 72 hours after a single injection of CCl_4_, however, PDGFRα-positive cells have a higher density around central veins at areas of hepatocyte injury (Figure 4E). We found that after six weeks of twice weekly CCl_4_ injections, PDGFRα positive cells are detected around and between portal veins, where fibrotic bands form (Figure 4F). As PDGFRβ is expressed in quiescent and activated HSCs [38], we next determined whether both PDGFRs are expressed in the same cell type. PDGFRα positive cells in chronic CCl_4_ injected *Pdgfrα^WT/nGFP^*mouse livers (Figure 5A, D) co-localize with PDGFRβ immunoreactive cells (Figure 5B, E), indicating that activated HSCs express both receptors (Figure 5C, F) after chronic CCl_4_ injection.

Our data from human tissue using IHC (Figure 1) and from mice using a GFP reporter (Figure 5) indicated that HSCs are the predominant liver cell type that expresses PDGFRα. In order to confirm these findings, we stained for specific liver cell epitopes using IF in combination with nuclear-GFP expression in *Pdgfrα^WT/nGFP^*mice [20]. Images of livers from *Pdgfrα^WT/nGFP^*mice indicate that PDGFRα and PDGFRβ co-localize in the same cells (Figure 5, Figure S2A), and that these cells also express desmin (Figure S2B) and cellular retinol binding protein 1 (CRBP-1, Figure S2C), proteins expressed in HSCs. Furthermore, GFP is not detected in Kupffer cells that express F4/80 expression (Figure S2D), or endothelial cells as identified by CD31 expression (Figure S2E). Hepatocytes, identified morphologically by fluorescence as described [39], were also negative for GFP (Figure S2F). Taken together, these data suggest that HSCs are the primary liver cell type which express PDGFRα and PDGFRβ.

### Reducing PDGFRα Expression Reduces Fibrosis in Mice

**Figure 6:**
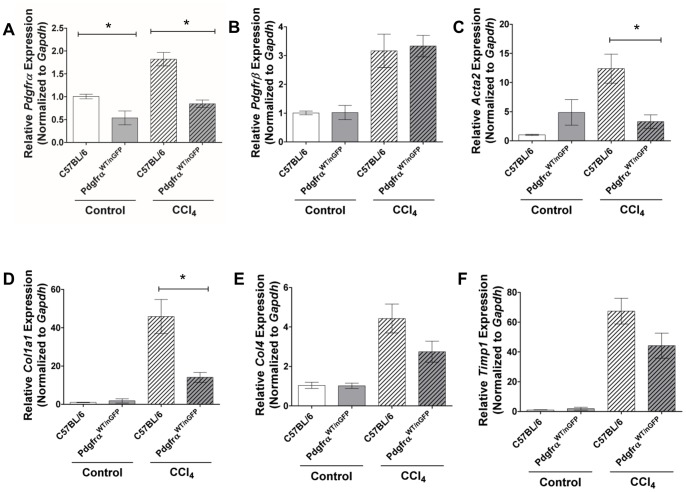
Compared to C57BL/6 mice, chronically CCl_4_ injured *Pdgfrα^WT/nGFP^* mice have reduced transcription of fibrotic genes. Livers from *Pdgfrα^WT/nGFP^* and C57BL/6 mice that were either uninjured (controls) or treated for 4 weeks with CCl_4_ were used to prepare total liver RNA. A) Compared to C57BL/6 mice, expression of *Pdgfrα* is decreased in *Pdgfrα^WT/nGFP^* mice in uninjured mice and after chronic CCl_4_. B) Expression of *Pdgfrβ* does not differ between C57BL/6 and *Pdgfrα^WT/nGFP^* mice. C) Expression of *Acta2* is increased in uninjured *Pdgfrα^WT/nGFP^* mice compared to C57BL/6 and decreased after chronic CCl_4_ between *Pdgfrα^WT/nGFP^* and C57BL/6 mice. D) Expression of *Col1a1* is similar in uninjured *Pdgfrα^WT/nGFP^* mice compared to C57BL/6 and decreased after chronic CCl_4_ between *Pdgfrα^WT/nGFP^* and C57BL/6 mice. E) Expression of *Col4* is similar in *Pdgfrα^WT/nGFP^* mice compared to C57BL/6 in both uninjured and chronic CCl_4_ injected mice. F) Expression of *Timp1* is increased to a similar level in both genotypes. Samples were processed as described in figure 2. Values are represented as means with SEM, and were analyzed by Mann-whitney non-parametric U test * = p<0.05, n = 3–6 mice per time point.

After confirming that *Pdgfrα* expression increases with CCl_4_-induced liver injury, and that PDGFRα-positive HSCs are activated by CCl_4_ exposure, we utilized *Pdgfrα^WT/nGFP^*mice to evaluate the functional significance of *Pdgfrα* expression during liver fibrosis. *Pdgfrα^WT/nGFP^*mice, which are heterozygous for *Pdgfrα* expression, are phenotypically normal [20] and have normal liver architecture (Figure 4A). Uninjured and CCl_4_-injected *Pdgfrα^WT/nGFP^* and C57BL/6 mice were analyzed for transcriptional changes in genes associated with chronic liver injury. At baseline and after 4 weeks of twice weekly injections of CCl_4_, *Pdgfrα* expression is decreased in *Pdgfrα^WT/nGFP^* mice compared to C57BL/6 mice (Figure 6A). *Pdgfrβ* expression was equivalently expressed in both genotypes of uninjured and chronic CCl_4_ injected mice (Figure 6B). Expression of smooth muscle α-actin (*Acta2*), an epitope that is upregulated when HSCs are activated [40], [41], increased when C57BL/6 mice were injected with CCl_4_ for 4 weeks, but *Pdgfrα^WT/nGFP^* mice had greatly reduced *Acta2* expression after chronic liver injury (Figure 6C). Fibrillar collagen 1a1 (*Col1a1*) expression was equivalent in uninjured mice of the two genotypes, but significantly reduced in chronically injured *Pdgfrα^WT/nGFP^* mice (Figure 6D). Conversely, collagen 4 (*Col4,* Figure 6E) and tissue inhibitor of metalloproteinase 1 (*Timp1,* Figure 6F) expression was increased after chronic CCl_4_ exposure, but was not significantly reduced in *Pdgfrα^WT/nGFP^* mice compared to wild type mice.

**Figure 7:**
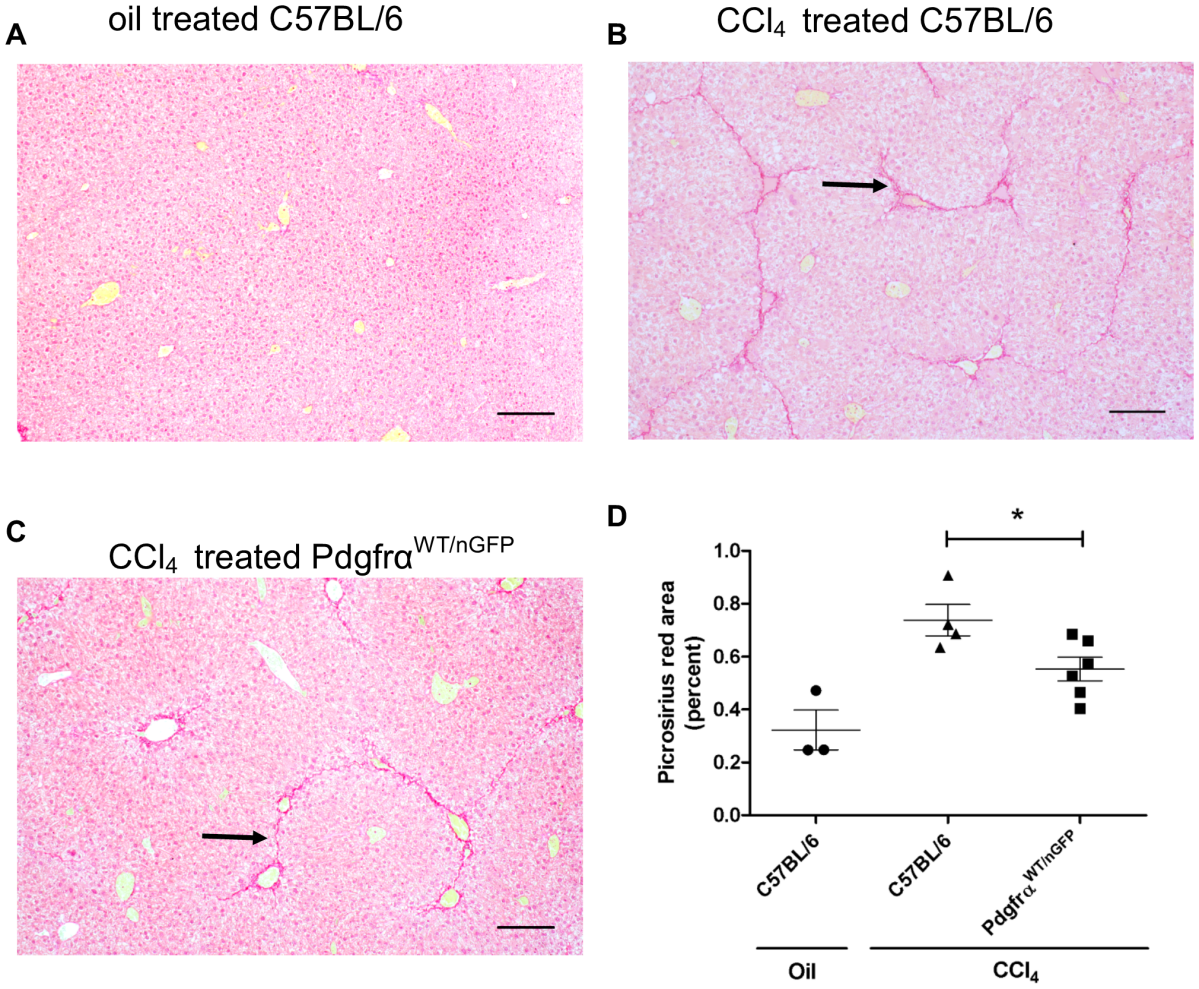
Chronically injured *Pdgfrα^WT/nGFP^* mice have less collagen deposition than C57BL/6 mice. Mice were injected with olive oil or CCl_4_ twice weekly for 4 weeks and collagen was detected in liver tissue by picrosirius red staining. A) Liver from a mouse injected with oil shows little collagen deposition. B) C57BL/6 mice develop fibrosis (arrow) after 4 weeks of CCl_4_ injections. C) *Pdgfrα^WT/nGFP^* mice develop less fibrosis (arrow) than C57BL/6 mice after 4 weeks of CCl_4_ injections. D) Quantification of picrosirius red positive area. Values are represented as means with SEM, and were analyzed by Mann-Whitney non-parametric U test * = p<0.05, n = 3–6 mice per time point. Scale bars are 100 μm.

Reduced mRNA expression of *Col1a1* in *Pdgfrα^WT/nGFP^* mice after chronic CCl_4_ injection was accompanied by a reduction in liver fibrosis, as assessed by picrosirius red staining, a histochemical assay for tissue fibrosis. C57BL/6 mice injected with vehicle for 4 weeks had little to no fibrosis (Figure 7A, D), but developed periportal fibrosis after 4 weeks of twice weekly CCl_4_ injections (Figure 7B, D). Significantly less collagen was deposited in chronically injured *Pdgfrα^WT/nGFP^* mice (Figure 7C, D). These results demonstrate that liver fibrosis in response to chronic CCl_4_ injection is dependent on normal expression of PDGFRα, and are consistent with the hypothesis that liver fibrosis is regulated in part by PDGFRα ligands.

## Discussion

PDGFRs stimulate proliferation, migration, and survival of mesenchymal cells, and increased activation of PDGFRs leads to organ fibrosis [21], [42]. Elevated expression of PDGFRs is associated with liver fibrosis and cirrhosis, so we sought to determine whether PDGFRα regulates liver fibrogenesis using mice that have one allele of *Pdgfrα* (*Pdgfrα^WT/nGFP^*). We found that mice with decreased *Pdgfrα* expression have less liver fibrosis after chronic CCl_4_ injury. In addition, and consistent with the notion that PDGFRα regulates the liver’s response to injury, patients with liver disease have elevated expression of PDGFRα and PDGFRβ. In conjunction, GFP localization in *Pdgfrα^WT/nGFP^* mice indicates that PDGFRα-positive HSCs migrate to sites of injury following CCl_4_ injection. These data all suggest that PDGFRα is involved in the activation of HSCs after hepatocyte injury.

PDGFRs are thought to play a central role in activating HSCs and promoting liver fibrosis and cirrhosis [33], [34], [43]; whether PDGFRα and PDGFRβ play independent roles in fibrogenesis is not known. We and others observe that *Pdgfrβ* expression increases in WT mice after acute liver injury by CCl_4_, implicating PDGFRβ in HSC activation. Thus it is surprising that mice which systemically express a hyperactive PDGFRβ allele do not develop more liver fibrosis than WT mice after 4 weeks of CCl_4_ injections [44]. Our data indicate that hepatocyte injury induces *Pdgfrα* expression above uninjured liver in both mice and humans, corroborating previously published studies [34], [37]. Our results demonstrate that expression of *Pdgfrα* and *Pdgfrβ* are both increased after chronic CCl_4_ liver injury, while reducing *Pdgfrα* copy number reduces *Pdgfrα* expression but not *Pdgfrβ* expression in *Pdgfrα^WT/nGFP^* mice. Reduced *Pdgfrα* expression in *Pdgfrα^WT/nGFP^* mice corrrelates with significantly reduced *Col1a1* and *Acta2* expression, as well as reduced picrosirius red staining, even though *Pdgfrβ* expression remains elevated. PDGFRα and PDGFRβ appear to affect HSCs differentially, despite being co-localized in the same liver cell type. Further studies will be necessary to dissect the receptor-specific contributions of PDGF signaling pathways in HSCs and in liver fibrosis.

Small perturbations in the PDGF signaling pathway, whether due to changes in expression of ligand or receptor, appear to have a large impact on specific diseases. Support for this notion is found in genetic evidence from rodents, which suggests that small changes in PDGFR activity *in vivo* are capable of significantly affecting a cell’s function. For example in development, chimerism studies show that both *Pdgfrβ^+/−^* and *Pdgfrα^+/−^* embryonic stem cells are deficient in contributing cells to the embryo [15], [45], and adult mice have a decreased number of progenitor cells in mice heterozygous *Pdgfrs* [46], [47]. Heterozygous *Pdgfrα* mice have been bred to mice with mutations in PDGF ligands [48] or mutations in immediate early genes directly downstream of PDGFRα [49], resulting in additive effects. However, deletion of one allele of *Pdgfrα* and the resultant heterozygosity does not affect development [15], [20]. These studies suggest that a single copy of *Pdgfrα* is usually sufficient for development, although under certain circumstances two alleles of *Pdgfrα* are required. In the current study, we found that *Pdgfrα^WT/nGFP^* mice have reduced fibrosis and reduced expression of the profibrotic genes *Acta2* and *Col1a1* after chronic CCl_4_ injury. Our data indicate that in chronic liver injury, PDGFRα plays a critical role in the development of fibrosis, but that other pathways also contribute to fibrogenesis. Expression of *Col4* and *Timp1* were reduced in *Pdgfrα^WT/nGFP^* mice but not to a significant extent, suggesting that expression of these genes could be more reliant on PDGFRα independent pathways, or heterogeneity in populations of HSCs.

We also sought to better define the role of PDGFRα in liver fibrosis by utilizing both human specimens and mouse models. Using a variety of experimental approaches, increased PDGFRα was seen in cirrhotic human livers and in mice with chemically-induced liver fibrosis. Although no preclinical rodent model fully recapitulates human liver fibrosis, there appears to be comparable molecular pathophysiology between humans and mice. We chose to utilize a knock-in mouse model expressing nuclear-GFP driven by the *Pdgfrα* promoter in order to discriminate between cells located in close proximity to each other, specifically different NPC populations in liver sinusoids [20]. We did not observe nuclear-GFP expression in hepatocytes, Kupffer cells, or LSECs, thus we conclude that the majority of PDGFRα is expressed in HSCs in the mouse liver, consistent with our observation that human liver specimens express PDGFRα primarily in NPCs. Our IHC data are further supported by data from the Human Protein Atlas (http://www.proteinatlas.org/ENSG00000134853/cancer), which demonstrates that NPCs are positive for PDGFRα by IHC in both normal liver and HCC [50].

We and others posit that selectively targeting PDGFRα in liver fibrosis and cirrhosis could reduce the proliferation, migration, and survival of the activated HSCs cells that contribute to collagen deposition. Therapeutic blockade of PDGFRα signaling may have a broad impact in the treatment of liver fibrosis, as four of the five PDGF ligand dimers, PDGF-AA, -AB, -BB, and –CC, bind and activate PDGFRα [12]. Targeting PDGFRβ, on the other hand, would completely inhibit only signal transduction induced by PDGF-DD, and could disrupt necessary functions of PDGFRβ in the liver. Targeting both PDGFRs with multi-kinase inhibitors, such as imatinib or sorafenib, leads to severe off target effects [51], [52]. The breadth of multi-kinase inhibitor activity thus likely leads to inhibition of beneficial signal transduction, either via PDGFRβ or other kinases. In summary, our data suggest that PDGFRα has a specific role in liver fibrosis in mice and in humans, and suggest that further mechanistic evaluation of PDGFRα function in the liver has the potential to uncover new anti-fibrotic therapies.

## Supporting Information

Figure S1 **Expression of PDGFRs in human liver tumor and non-tumor tissue.** An immunoblot of PDGFRα detects PDGFRα in non-tumor tissue while an immunoblot for PDGFRβ shows protein in both tumor and surrounding tissue. Albumin was used as a loading control.
(TIF)

Figure S2 **HSCs express PDGFRα.** PDGFRα-driven nuclear GFP is expressed in liver cells that are immunoreactive for common HSC markers: A) PDGFRβ (red), B) Desmin (red), and C) cellular retinol binding protein 1 (CRBP-1) (red). PDGFRα and CRBP-1 are not expressed in cells that stain for D) the Kupffer cell marker F4/80 (blue) or E) the endothelial cell marker CD31(blue). F) PDGFRα positive cells (green) are distinct from hepatocytes (yellow). Scale bars are 10 μm.
(TIF)

Table S1 **Antibodies used in this study.**
(DOCX)

Table S2 **Human and mouse hepatocyte and stellate cell lines used in this study.** *references as PMID number.
(DOCX)

Table S3 **Primers used for real time analysis.**
(DOCX)

Table S4 **Summary of PDGFRα and PDGFRβ immunoreactivity in human liver specimens.** Resected liver specimens with HCCs were formalin-fixed, paraffin embedded, and evaluated for the presence of cirrhosis and HCC. IHC for PDGFRα and PDGFRβ was performed as described in Materials and Methods. Relative staining intensity is indicated as weak (+), moderate (++), strong (+++), or absent (0).
(DOCX)

Table S5 **Immunoblot detection of PDGFR expression in macroscopically dissected human tumors and surrounding liver.** HCCs (Tumor) and surrounding liver (Non-Tumor) were macrodissected from patients, frozen, and processed for immunoblot analysis as described in Materials and Methods. Intensity is indicated as present (+) or absent (0).
(DOCX)

Table S6 **PDGF stimulates proliferation^1^ in stellate cell lines, but not primary hepatocytes or hepatoma cell lines.** *^1^*Cell proliferation was measured by DNA synthesis using tritiated thymidine incorporation [28]. The data is the average of three different experiments that were each done in triplicates. Fold change represents the increase when compared to unstimulated cells for each cell line. *^2^“*Positive control” indicates that DNA synthesis was stimulated in each cell line or primary culture with a growth factor previously reported to simulate proliferation. Growth factors used for each cell and the concentrations are as follows: mouse hepatocytes, EGF (20 ng/mL); AML12 cells, EGF (20 ng/mL); NMH cells, HB-EGF (20 ng/mL); rat stellate cells (2G), 1% fetal calf serum; human stellate cells (LX-2), 1% FCS; SK-Hep (human hepatoma cells of endothelial origin), 10% fetal calf sera.
(DOCX)

## References

[1] Starr SP, Raines D (2011) Cirrhosis: diagnosis, management, and prevention. Am Fam Physician 84: 1353–1359. 22230269

[2] Bataller R, Brenner DA (2005) Liver fibrosis. J Clin Invest 115: 209–218. 15690074 10.1172/JCI24282 PMC546435

[3] Hernandez-Gea V, Friedman SL (2011) Pathogenesis of liver fibrosis. Annu Rev Pathol 6: 425–456. 21073339 10.1146/annurev-pathol-011110-130246

[4] Pierce GF, Tarpley JE, Tseng J, Bready J, Chang D, et al (1995) Detection of platelet-derived growth factor (PDGF)-AA in actively healing human wounds treated with recombinant PDGF-BB and absence of PDGF in chronic nonhealing wounds. J Clin Invest 96: 1336–1350. 7657809 10.1172/JCI118169 PMC185756

[5] Olson LE, Soriano P (2009) Increased PDGFRalpha activation disrupts connective tissue development and drives systemic fibrosis. Dev Cell 16: 303–313. 19217431 10.1016/j.devcel.2008.12.003 PMC2664622

[6] Chong JJ, Reinecke H, Iwata M, Torok-Storb B, Stempien-Otero A, et al (2013) Progenitor cells identified by PDGFR-alpha expression in the developing and diseased human heart. Stem Cells Dev 22: 1932–1943. 23391309 10.1089/scd.2012.0542 PMC3685392

[7] Antoniades HN, Bravo MA, Avila RE, Galanopoulos T, Neville-Golden J, et al (1990) Platelet-derived growth factor in idiopathic pulmonary fibrosis. J Clin Invest 86: 1055–1064. 2170444 10.1172/JCI114808 PMC296832

[8] Floege J, Eitner F, Alpers CE (2008) A new look at platelet-derived growth factor in renal disease. J Am Soc Nephrol 19: 12–23. 18077793 10.1681/ASN.2007050532

[9] Zymek P, Bujak M, Chatila K, Cieslak A, Thakker G, et al (2006) The role of platelet-derived growth factor signaling in healing myocardial infarcts. J Am Coll Cardiol 48: 2315–2323. 17161265 10.1016/j.jacc.2006.07.060

[10] Abdollahi A, Li M, Ping G, Plathow C, Domhan S, et al (2005) Inhibition of platelet-derived growth factor signaling attenuates pulmonary fibrosis. J Exp Med 201: 925–935. 15781583 10.1084/jem.20041393 PMC2213091

[11] Chen YT, Chang FC, Wu CF, Chou YH, Hsu HL, et al (2011) Platelet-derived growth factor receptor signaling activates pericyte-myofibroblast transition in obstructive and post-ischemic kidney fibrosis. Kidney Int 80: 1170–1181. 21716259 10.1038/ki.2011.208

[12] Andrae J, Gallini R, Betsholtz C (2008) Role of platelet-derived growth factors in physiology and medicine. Genes Dev 22: 1276–1312. 18483217 10.1101/gad.1653708 PMC2732412

[13] Hoch RV, Soriano P (2003) Roles of PDGF in animal development. Development 130: 4769–4784. 12952899 10.1242/dev.00721

[14] Soriano P (1994) Abnormal kidney development and hematological disorders in PDGF beta-receptor mutant mice. Genes Dev 8: 1888–1896. 7958864 10.1101/gad.8.16.1888

[15] Soriano P (1997) The PDGF alpha receptor is required for neural crest cell development and for normal patterning of the somites. Development 124: 2691–2700. 9226440 10.1242/dev.124.14.2691

[16] Mellgren AM, Smith CL, Olsen GS, Eskiocak B, Zhou B, et al (2008) Platelet-derived growth factor receptor beta signaling is required for efficient epicardial cell migration and development of two distinct coronary vascular smooth muscle cell populations. Circ Res 103: 1393–1401. 18948621 10.1161/CIRCRESAHA.108.176768 PMC2757035

[17] Olson LE, Soriano P (2011) PDGFRβ signaling regulates mural cell plasticity and inhibits fat development. Dev Cell 20: 815–826. 21664579 10.1016/j.devcel.2011.04.019 PMC3121186

[18] Chen H, Gu X, Liu Y, Wang J, Wirt SE, et al (2011) PDGF signalling controls age-dependent proliferation in pancreatic β-cells. Nature 478: 349–355. 21993628 10.1038/nature10502 PMC3503246

[19] Tallquist MD, Soriano P (2003) Cell autonomous requirement for PDGFRalpha in populations of cranial and cardiac neural crest cells. Development 130: 507–518. 12490557 10.1242/dev.00241

[20] Hamilton TG, Klinghoffer RA, Corrin PD, Soriano P (2003) Evolutionary divergence of platelet-derived growth factor alpha receptor signaling mechanisms. Mol Cell Biol 23: 4013–4025. 12748302 10.1128/MCB.23.11.4013-4025.2003 PMC155222

[21] Bonner JC (2004) Regulation of PDGF and its receptors in fibrotic diseases. Cytokine Growth Factor Rev 15: 255–273. 15207816 10.1016/j.cytogfr.2004.03.006

[22] Iwayama T, Olson LE (2013) Involvement of PDGF in fibrosis and scleroderma: recent insights from animal models and potential therapeutic opportunities. Curr Rheumatol Rep 15: 304. 23307576 10.1007/s11926-012-0304-0 PMC5570472

[23] Yeh MM, Larson AM, Campbell JS, Fausto N, Rulyak SJ, et al (2007) The expression of transforming growth factor-alpha in cirrhosis, dysplastic nodules, and hepatocellular carcinoma: an immunohistochemical study of 70 cases. Am J Surg Pathol 31: 681–689. 17460450 10.1097/PAS.0b013e31802ff7aa

[24] Campbell JS, Johnson MM, Bauer RL, Hudkins KL, Gilbertson DG, et al (2007) Targeting stromal cells for the treatment of platelet-derived growth factor C-induced hepatocellular carcinogenesis. Differentiation 75: 843–852. 17999742 10.1111/j.1432-0436.2007.00235.x

[25] Junqueira LC, Bignolas G, Brentani RR (1979) Picrosirius staining plus polarization microscopy, a specific method for collagen detection in tissue sections. Histochem J 11: 447–455. 91593 10.1007/BF01002772

[26] Campbell JS, Riehle KJ, Brooling JT, Bauer RL, Mitchell C, et al (2006) Proinflammatory cytokine production in liver regeneration is Myd88-dependent, but independent of Cd14, Tlr2, and Tlr4. J Immunol 176: 2522–2528. 16456013 10.4049/jimmunol.176.4.2522

[27] Wright JH, Johnson MM, Shimizu-Albergine M, Bauer RL, Hayes BJ, et al.. (2013) Paracrine activation of hepatic stellate cells in platelet-derived growth factor C transgenic mice: Evidence for stromal induction of hepatocellular carcinoma. Int J Cancer. 10.1002/ijc.28421 PMC3876966 23929039

[28] Argast GM, Campbell JS, Brooling JT, Fausto N (2004) Epidermal growth factor receptor transactivation mediates tumor necrosis factor-induced hepatocyte replication. J Biol Chem 279: 34530–34536. 15199050 10.1074/jbc.M405703200

[29] Thieringer F, Maass T, Czochra P, Klopcic B, Conrad I, et al (2008) Spontaneous hepatic fibrosis in transgenic mice overexpressing PDGF-A. Gene 423: 23–28. 18598744 10.1016/j.gene.2008.05.022

[30] Czochra P, Klopcic B, Meyer E, Herkel J, Garcia-Lazaro JF, et al (2006) Liver fibrosis induced by hepatic overexpression of PDGF-B in transgenic mice. J Hepatol 45: 419–428. 16842882 10.1016/j.jhep.2006.04.010

[31] Campbell JS, Hughes SD, Gilbertson DG, Palmer TE, Holdren MS, et al (2005) Platelet-derived growth factor C induces liver fibrosis, steatosis, and hepatocellular carcinoma. Proc Natl Acad Sci U S A 102: 3389–3394. 15728360 10.1073/pnas.0409722102 PMC552940

[32] Ikura Y, Morimoto H, Ogami M, Jomura H, Ikeoka N, et al (1997) Expression of platelet-derived growth factor and its receptor in livers of patients with chronic liver disease. J Gastroenterol 32: 496–501. 9250897 10.1007/BF02934089

[33] Wong L, Yamasaki G, Johnson RJ, Friedman SL (1994) Induction of beta-platelet-derived growth factor receptor in rat hepatic lipocytes during cellular activation in vivo and in culture. J Clin Invest 94: 1563–1569. 7929832 10.1172/JCI117497 PMC295310

[34] Pinzani M, Milani S, Herbst H, DeFranco R, Grappone C, et al (1996) Expression of platelet-derived growth factor and its receptors in normal human liver and during active hepatic fibrogenesis. Am J Pathol 148: 785–800. 8774134 PMC1861723

[35] Tiribelli C, Melato M, Crocè LS, Giarelli L, Okuda K, et al (1989) Prevalence of hepatocellular carcinoma and relation to cirrhosis: comparison of two different cities of the world–Trieste, Italy, and Chiba, Japan. Hepatology 10: 998–1002. 2555298 10.1002/hep.1840100618

[36] Weber LW, Boll M, Stampfl A (2003) Hepatotoxicity and mechanism of action of haloalkanes: carbon tetrachloride as a toxicological model. Crit Rev Toxicol 33: 105–136. 12708612 10.1080/713611034

[37] Borkham-Kamphorst E, Kovalenko E, van Roeyen CR, Gassler N, Bomble M, et al (2008) Platelet-derived growth factor isoform expression in carbon tetrachloride-induced chronic liver injury. Lab Invest 88: 1090–1100. 18663351 10.1038/labinvest.2008.71

[38] Breitkopf K, Roeyen C, Sawitza I, Wickert L, Floege J, et al (2005) Expression patterns of PDGF-A, -B, -C and -D and the PDGF-receptors alpha and beta in activated rat hepatic stellate cells (HSC). Cytokine 31: 349–357. 16039137 10.1016/j.cyto.2005.06.005

[39] Johnson S, Rabinovitch P (2012) Ex vivo imaging of excised tissue using vital dyes and confocal microscopy. Curr Protoc Cytom Chapter 9: Unit 9.39. 10.1002/0471142956.cy0939s61 PMC3401092 22752953

[40] Rockey DC, Boyles JK, Gabbiani G, Friedman SL (1992) Rat hepatic lipocytes express smooth muscle actin upon activation in vivo and in culture. J Submicrosc Cytol Pathol 24: 193–203. 1600511

[41] Yamaoka K, Nouchi T, Marumo F, Sato C (1993) Alpha-smooth-muscle actin expression in normal and fibrotic human livers. Dig Dis Sci 38: 1473–1479. 8344103 10.1007/BF01308606

[42] Heldin CH, Westermark B (1999) Mechanism of action and in vivo role of platelet-derived growth factor. Physiol Rev 79: 1283–1316. 10508235 10.1152/physrev.1999.79.4.1283

[43] Friedman SL, Arthur MJ (1989) Activation of cultured rat hepatic lipocytes by Kupffer cell conditioned medium. Direct enhancement of matrix synthesis and stimulation of cell proliferation via induction of platelet-derived growth factor receptors. J Clin Invest 84: 1780–1785. 2556445 10.1172/JCI114362 PMC304055

[44] Krampert M, Heldin CH, Heuchel RL (2008) A gain-of-function mutation in the PDGFR-beta alters the kinetics of injury response in liver and skin. Lab Invest 88: 1204–1214. 18762776 10.1038/labinvest.2008.81

[45] Crosby JR, Seifert RA, Soriano P, Bowen-Pope DF (1998) Chimaeric analysis reveals role of Pdgf receptors in all muscle lineages. Nat Genet 18: 385–388. 9537425 10.1038/ng0498-385

[46] McKinnon RD, Waldron S, Kiel ME (2005) PDGF alpha-receptor signal strength controls an RTK rheostat that integrates phosphoinositol 3′-kinase and phospholipase Cgamma pathways during oligodendrocyte maturation. J Neurosci 25: 3499–3508. 15814780 10.1523/JNEUROSCI.5049-04.2005 PMC6725367

[47] Bell RD, Winkler EA, Sagare AP, Singh I, LaRue B, et al (2010) Pericytes control key neurovascular functions and neuronal phenotype in the adult brain and during brain aging. Neuron 68: 409–427. 21040844 10.1016/j.neuron.2010.09.043 PMC3056408

[48] Andrae J, Ehrencrona H, Gallini R, Lal M, Ding H, et al.. (2013) Analysis of mice lacking the heparin-binding splice isoform of PDGF-A. Mol Cell Biol. 10.1128/MCB.00749-13 PMC3811674 23938297

[49] Schmahl J, Raymond C, Soriano P (2007) PDGF signaling specificity is mediated through multiple immediate early genes. Nat Genet 39: 52–60. 17143286 10.1038/ng1922

[50] Uhlen M, Oksvold P, Fagerberg L, Lundberg E, Jonasson K, et al (2010) Towards a knowledge-based Human Protein Atlas. Nat Biotechnol 28: 1248–1250. 21139605 10.1038/nbt1210-1248

[51] Teo YL, Ho HK, Chan A (2013) Risk of tyrosine kinase inhibitors-induced hepatotoxicity in cancer patients: A meta-analysis. Cancer Treat Rev 39: 199–206. 23099278 10.1016/j.ctrv.2012.09.004

[52] Hasinoff BB, Patel D (2010) The lack of target specificity of small molecule anticancer kinase inhibitors is correlated with their ability to damage myocytes in vitro. Toxicol Appl Pharmacol 249: 132–139. 20832415 10.1016/j.taap.2010.08.026

