# Supplementary figures and images for: Activation of Platelet-Derived Growth Factor Receptor Alpha Contributes to Liver Fibrosis
Source: PLoS One. 2014 Mar 25;9(3):e92925. doi: 10.1371/journal.pone.0092925 (PMC3965491; doi:10.1371/journal.pone.0092925)

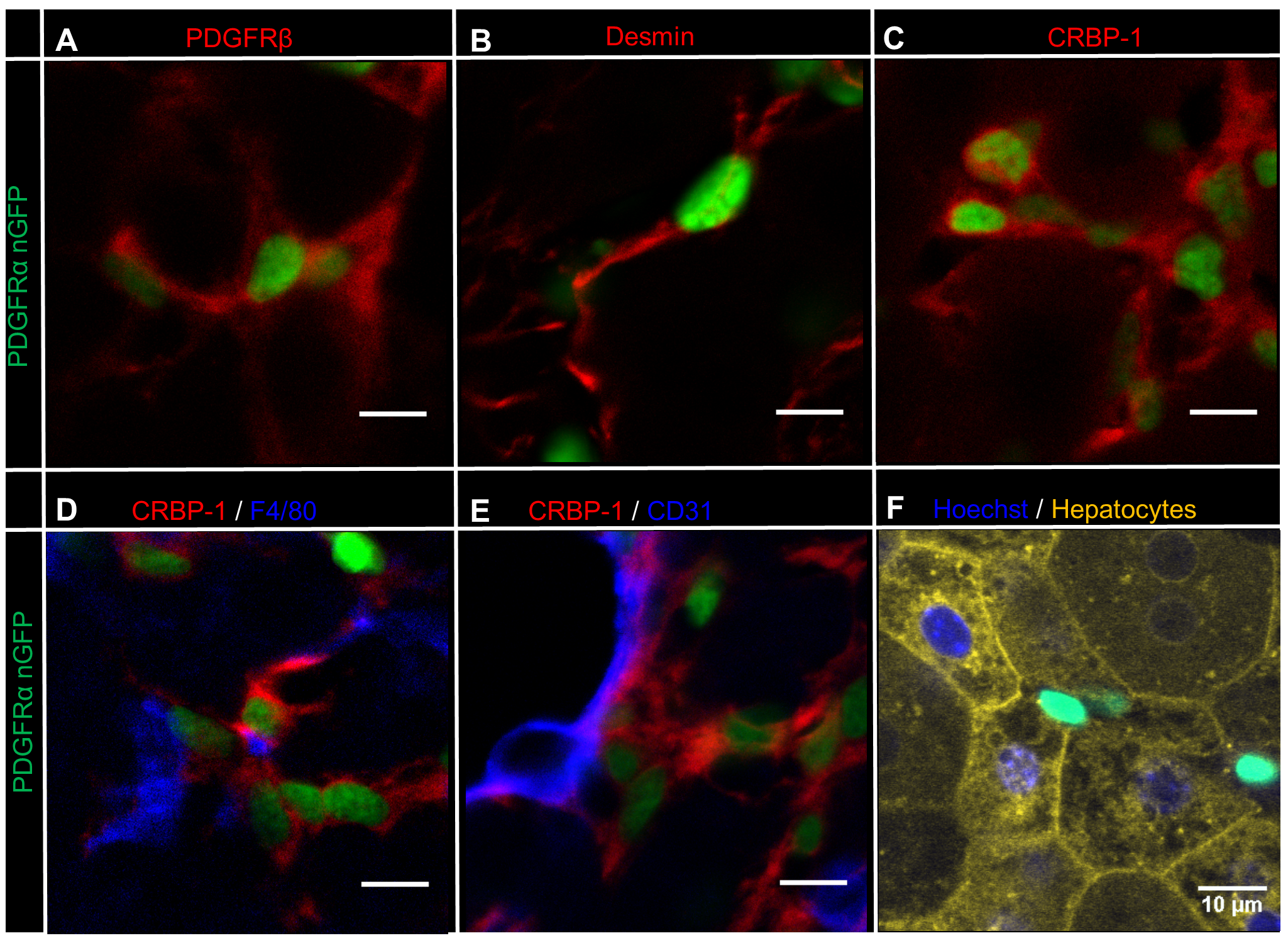

Supplement: Figure S2 — HSCs express PDGFRα. PDGFRα-driven nuclear GFP is expressed in liver cells that are immunoreactive for common HSC markers: A) PDGFRβ (red), B) Desmin (red), and C) cellular retinol binding protein 1 (CRBP-1) (red). PDGFRα and CRBP-1 are not expressed in cells that stain for D) the Kupffer cell marker F4/80 (blue) or E) the endothelial cell marker CD31(blue). F) PDGFRα positive cells (green) are distinct from hepatocytes (yellow). Scale bars are 10 μm. (TIF) [file pone.0092925.s002.tif]
