# Supplementary material for: Activation of Platelet-Derived Growth Factor Receptor Alpha Contributes to Liver Fibrosis
Source: PLoS One. 2014 Mar 25;9(3):e92925. doi: 10.1371/journal.pone.0092925 (PMC3965491; doi:10.1371/journal.pone.0092925)
Supplement: Table S1 — Antibodies used in this study. (DOCX) [file pone.0092925.s003.docx]

**Table S1: Antibodies used in this study**

|  | | | |
| --- | --- | --- | --- |
| **Antigen** | **Manufacturer** | **catalog number** | **Use** |
| PDGFRα | Cell Signaling Technologies | 3164 | Western Blot, Immunohistochemistry |
| PDGFRα | Santa Cruz Biotechnology | sc-338 | Western Blot |
| PDGFRβ | Epitomics | APB5 | Immunofluorescence |
| PDGFRβ | Cell Signaling Technologies | 3169 | Western Blot, Immunohistochemistry |
| Desmin | Epitomics | 1466-1 | Immunofluorescence |
| CRBP-1 | Santa Cruz Biotechnology | sc-30106 | Immunofluorescence |
| F4/80 | Abd Serotec | MCA497A647 | Immunofluorescence |
| CD-31 | BD Biosciences | 553370 | Immunofluorescence |
| GAPDH | Genscript | A00192 | Western Blot |
| Albumin | MP Bio | 55235 | Western Blot |
