# Supplementary material for: Activation of Platelet-Derived Growth Factor Receptor Alpha Contributes to Liver Fibrosis
Source: PLoS One. 2014 Mar 25;9(3):e92925. doi: 10.1371/journal.pone.0092925 (PMC3965491; doi:10.1371/journal.pone.0092925)
Supplement: Table S2 — Human and mouse hepatocyte and stellate cell lines used in this study. *references as PMID number. (DOCX) [file pone.0092925.s004.docx]

**Table S2: Human and mouse hepatocyte and stellate cell lines used in this study**

| **Human Stellate Cells** | | | | | | |
| --- | --- | --- | --- | --- | --- | --- |
| **Cell line** | **Origin** | | **Source** | | **PMID** | |
| LX-1 | SV40 large T immortalized hepatic stellate cell | | Dr. S. L. Friedman | | 15591520 | |
| LX-2 | low serum adapted subclone of LX-1 | | Dr. S. L. Friedman | | 15591520 | |
|  |  |  | |  | |  |
| **Rat Stellate Cells** | | | | | |  |
| **Cell line** | **Origin** | **Source** | | **PMID** | |  |
| CFSC-2G | CCl_4_ treated rat |  | | 8394478 | |  |

| **Human Hepatocytes** | | | |
| --- | --- | --- | --- |
| **Cell line** | **Origin** | **Source** | **PMID** |
| Hep 3B | 8 year old male hepatocellular carcinoma hepatitis B positive | ATCC: HB-8064 | 233137 |
|  |  |  |  |
| Hep G2 | 15 year old male hepatocellular carcinoma hepatitis B negative | ATCC: HB-8065 | 233137 |
|  |  |  |  |
| HuH-7 | 57 year old male differentiated hepatocellular carcinoma (well differentiated) hepatitis B negative | JCRB0403 | 6286115 |
|  |  |  |  |
|  |  |  |  |
|  |  |  |  |
| HH4 | Normal human hepatocytes immortalized by HPV E6/E7 | Dr. N. Fausto | 17991716 |
| SK-Hep cells | Hepatocellular carcinoma | ATCC: HTB-52 | 327080 |
|  |  |  |  |
| **Mouse Hepatocytes** | | | |
| **Cell line** | **Origin** | **Source** | **PMID** |
| AML12 | hTGFα transgene driven by MT1 | ATCC: CRL-2254 | 7904757 |
| NMH | Normal BALB/c liver |  | 7775596 |
