# Supplementary material for: Activation of Platelet-Derived Growth Factor Receptor Alpha Contributes to Liver Fibrosis
Source: PLoS One. 2014 Mar 25;9(3):e92925. doi: 10.1371/journal.pone.0092925 (PMC3965491; doi:10.1371/journal.pone.0092925)
Supplement: Table S3 — Primers used for real time analysis. (DOCX) [file pone.0092925.s005.docx]

**Table S3: Primers used for real time analysis.**

| **Taqman primers** | |
| --- | --- |
| **Target Gene** | **catalog number** |
| *PDGFRA* (human) | Hs00183486_m1 |
| *PDGFRB* (human) | Hs01019589_m1 |
| *18S* (human) | Hs99999901_s1 |
| *Gapdh* (mouse) | Mm99999915_g1 |
| *Pdgfra* (mouse) | Mm01211694_m1 |
| *Pdgfrb* (mouse) | Mm00435546_m1 |
| *Acta2* (mouse) | Mm01546133_m1 |
| *Col1a1* (mouse) | Mm00801666_g1 |
| *Col4* (mouse) | Mm01210125_m1 |
| *Timp1* (mouse) | Mm00441818_m1 |
