# Supplementary material for: Activation of Platelet-Derived Growth Factor Receptor Alpha Contributes to Liver Fibrosis
Source: PLoS One. 2014 Mar 25;9(3):e92925. doi: 10.1371/journal.pone.0092925 (PMC3965491; doi:10.1371/journal.pone.0092925)
Supplement: Table S4 — Summary of PDGFRα and PDGFRβ immunoreactivity in human liver specimens. Resected liver specimens with HCCs were formalin-fixed, paraffin embedded, and evaluated for the presence of cirrhosis and HCC. IHC for PDGFRα and PDGFRβ was performed as described in Materials and Methods. Relative staining intensity is indicated as weak (+), moderate (++), strong (+++), or absent (0). (DOCX) [file pone.0092925.s006.docx]

**Table S4: Summary of PDGFRα and PDGFRβ immunoreactivity in human liver specimens**

| Patient | Tumor present | Cirrhosis | Stromal PDGFRα | Stromal PDGFRβ | Other |
| --- | --- | --- | --- | --- | --- |
| 1 | no | yes | +++ | + |  |
| 5 | yes |  | ++ | + | No non-tumor |
| 9 | yes | no | + | N.D. |  |
| 13 | yes | yes | + | 0 |  |
| 14 | yes |  | + | + | No non-tumor |
| 15 | yes | yes | + | 0 |  |
| 16 | yes |  | N.D. | + |  |
| 17 | yes |  | N.D. | 0 |  |
| 18 | yes | yes | N.D. | 0 |  |
| 19 | yes |  | N.D. | 0 |  |
| 20 | yes | yes | + | 0 |  |
| 21 | yes | yes | +++ | 0 |  |
| 22 | no | yes | + | 0 |  |
| 23 | yes | no | ++ | + |  |
| 24 | yes |  | + |  |  |
| 25 | no | yes | + | 0 |  |
| 26 | yes | yes | + |  |  |
| 27 | yes | yes | + | + |  |
| 28 | yes |  | + | ++ | No non-tumor |
| 29 | yes | no | ++ | 0 |  |
| 30 | yes |  | + | + |  |
| 31 | yes | yes | + | + |  |
| 32 | yes | yes | + | + |  |
| 33 | yes | yes | + | N.D. |  |
| 34 | yes | yes | +++ | N.D. |  |
| 35 | yes | yes | + | N.D. |  |
| 36 | yes |  | + | N.D. |  |
| 37 | yes | yes | ++ | + |  |
| 38 | yes | no | + | + |  |
| 39 | yes | yes | + | ++ |  |
| 40 | yes | yes | + | + |  |
| 41 | yes | yes | +++ | + |  |
| 42 | yes | yes | + | + |  |
| 43 | yes | yes | +++ | 0 |  |
| 44 | no |  | + | + |  |
| 45 | yes |  | N/A |  | No non-tumor |
| 46 |  |  |  | + |  |
| 47 | yes |  |  | N.D. |  |
| 48 | yes | no | + | N.D. |  |
| 49 | yes | no |  | N.D. |  |
| 50 | yes | yes | +++ | N.D. |  |
| 51 | yes | no | 0 | N.D. |  |
| 52 | yes | no | 0 | N.D. |  |
| 53 | yes |  | +++ | N.D. |  |
