# Supplementary material for: Activation of Platelet-Derived Growth Factor Receptor Alpha Contributes to Liver Fibrosis
Source: PLoS One. 2014 Mar 25;9(3):e92925. doi: 10.1371/journal.pone.0092925 (PMC3965491; doi:10.1371/journal.pone.0092925)
Supplement: Table S5 — Immunoblot detection of PDGFR expression in macroscopically dissected human tumors and surrounding liver. HCCs (Tumor) and surrounding liver (Non-Tumor) were macrodissected from patients, frozen, and processed for immunoblot analysis as described in Materials and Methods. Intensity is indicated as present (+) or absent (0). (DOCX) [file pone.0092925.s007.docx]

**Table S5: Immunoblot detection of PDGFR expression in macroscopically dissected human tumors and surrounding liver**

| **Patient** | **Tissue** | **PDGFRα** | **PDGFRβ** |
| --- | --- | --- | --- |
| 1 | Non-tumor | 0 | + |
| 1 | Tumor | 0 | + |
| 2 | Non-tumor | + | + |
| 2 | Tumor | 0 | 0 |
| 3 | Non-tumor | 0 | + |
| 3 | Tumor | 0 | + |
| 4 | Non-tumor | + | + |
| 4 | Tumor | 0 | + |
| 5 | Non-tumor | 0 | 0 |
| 5 | Tumor | + | + |
| 6 | Non-tumor | 0 | 0 |
| 6 | Tumor | 0 | 0 |
| 7 | Non-tumor | 0 | 0 |
| 7 | Tumor | 0 | 0 |
| 8 | Non-tumor | 0 | 0 |
| 8 | Tumor | 0 | 0 |
| 9 | Non-tumor | + | + |
| 9 | Tumor | 0 | 0 |
| 10 | Non-tumor | 0 | 0 |
| 10 | Tumor | 0 | 0 |
| 11 | Non-tumor | + | 0 |
| 11 | Tumor | 0 | 0 |
| 12 | Non-tumor | 0 | 0 |
| 12 | Tumor | 0 | 0 |

.
