# Supplementary material for: Activation of Platelet-Derived Growth Factor Receptor Alpha Contributes to Liver Fibrosis
Source: PLoS One. 2014 Mar 25;9(3):e92925. doi: 10.1371/journal.pone.0092925 (PMC3965491; doi:10.1371/journal.pone.0092925)
Supplement: Table S6 — PDGF stimulates proliferation1 in stellate cell lines, but not primary hepatocytes or hepatoma cell lines. 1Cell proliferation was measured by DNA synthesis using tritiated thymidine incorporation [28]. The data is the average of three different experiments that were each done in triplicates. Fold change represents the increase when compared to unstimulated cells for each cell line. 2“Positive control” indicates that DNA synthesis was stimulated in each cell line or primary culture with a growth factor previously reported to simulate proliferation. Growth factors used for each cell and the concentrations are as follows: mouse hepatocytes, EGF (20 ng/mL); AML12 cells, EGF (20 ng/mL); NMH cells, HB-EGF (20 ng/mL); rat stellate cells (2G), 1% fetal calf serum; human stellate cells (LX-2), 1% FCS; SK-Hep (human hepatoma cells of endothelial origin), 10% fetal calf sera. (DOCX) [file pone.0092925.s008.docx]

**Table S6: PDGF stimulates proliferation^1^ in stellate cell lines, but not primary hepatocytes or hepatoma cell lines.**

|  | **PDGF-AA** | **PDGF-AB** | **PDGF-BB** | **PDGF-CC** | **Positive control2** |
| --- | --- | --- | --- | --- | --- |
| **Human stellate cells (LX-2)** | 3 fold increase | 3 fold increase | 3 fold increase | 3 fold increase | 3 fold increase |
| **Rat stellate cells**  **(2G)** | 5 fold increase | 5 fold increase | 5 fold increase | 5 fold increase | 10 fold increase |
| **Primary**  **hepatocytes (mouse)** | No stimulation | No stimulation | No stimulation | No stimulation | 2.5 fold increase |
| **AML12**  **hepatocytes**  **(mouse)** | No stimulation | No stimulation | No stimulation | No stimulation | 5 fold increase |
| **NMH**  **hepatocytes (mouse)** | No stimulation | No stimulation | No stimulation | No stimulation | 10 fold increase |
| **HH4**  **hepatocytes (human)** | No stimulation | No stimulation | No stimulation | No stimulation | 5 fold increase |
| **SK-Hep cells (human)** | No stimulation | No stimulation | No stimulation | No stimulation | 10 fold increase |
